# Effects of Neutralization on the Physicochemical, Mechanical, and Biological Properties of Ammonium-Hydroxide-Crosslinked Chitosan Scaffolds

**DOI:** 10.3390/ijms232314822

**Published:** 2022-11-26

**Authors:** Paola Hassibe Azueta-Aguayo, Martha Gabriela Chuc-Gamboa, Fernando Javier Aguilar-Pérez, Fernando Javier Aguilar-Ayala, Beatriz A. Rodas-Junco, Rossana Faride Vargas-Coronado, Juan Valerio Cauich-Rodríguez

**Affiliations:** 1 Facultad de Odontología, Universidad Autónoma de Yucatán, Mérida 97000, Mexico; 2 CONACYT—Facultad de Ingeniería Química, Universidad Autónoma de Yucatán, Mérida 97000, Mexico; 3 Unidad de Materiales, Centro de Investigación Científica de Yucatán, Calle 43 No. 130 x 32 y 34, Colonia Chuburná de Hidalgo, Mérida 97205, Mexico

**Keywords:** biopolymer, pH effect, tissue engineering scaffolds, mechanical properties, biomaterial interactions with mesenchymal stem cells

## Abstract

It has been reported that chitosan scaffolds, due to their physicochemical properties, stimulate cell proliferation in different tissues of the human body. This study aimed to determine the physicochemical, mechanical, and biological properties of chitosan scaffolds crosslinked with ammonium hydroxide, with different pH values, to better understand cell behavior depending on the pH of the biomaterial. Scaffolds were either neutralized with sodium hydroxide solution, washed with distilled water until reaching a neutral pH, or kept at alkaline pH. Physicochemical characterization included scanning electron microscopy (SEM), elemental composition (EDX), Fourier-transform infrared (FTIR) spectroscopy, Raman spectroscopy, thermogravimetric analysis (TGA), and mechanical testing. In vitro cytotoxicity was assessed via dental-pulp stem cells’ (DPSCs’) biocompatibility. The results revealed that the neutralized scaffolds exhibited better cell proliferation and morphology. It was concluded that the chitosan scaffolds’ high pH (due to residual ammonium hydroxide) decreases DPSCs’ cell viability.

## 1. Introduction

Tissue engineering is considered an important therapeutic tool in regenerative medicine; one of the crucial factors for tissue engineering is the scaffolds, which provide a platform for cells’ adhesion while allowing their growth [1,2]. Scaffolds are usually made from different materials, including ceramics and polymers. Natural polymers such as chitosan, gelatin, fibrin, collagen, and alginate are widely used as scaffolding materials alone or in combination with other materials [1,3,4].

Tissue engineering of dental and oral tissues has been used in recent years as a reliable option to treat different pathologies. This includes alveolar reconstruction, which is considered a complex and challenging procedure for maxillofacial and periodontal surgeons [5]. The crucial objective of this therapy is increase alveolar bone mass in patients who have suffered from bone loss because of various conditions, such as periodontal disease, aging, osteoporosis, trauma, neoplastic pathology, or reconstructive surgery [6]. In consequence, the search for new biomaterials that promote cell proliferation to replace bone defects via scaffolds based on biopolymers is considered an area of interest in the dental field [5,6].

Chitosan (CHT) is a biopolymer derived from the deacetylation of chitin, which is one of the most abundant polysaccharides in nature [7,8]. Chitosan is bioabsorbable, biodegradable, and has been shown to be slowly degraded, mainly by enzymes such as chitosanases and lysozymes [9]. The ability to modify factors such as pH, morphology, and viscosity makes chitosan an ideal biomaterial to be used in the oral cavity [10,11,12]. Furthermore, chitosan derivatives promote fibroblast proliferation, suggesting that chitosan does not have toxic effects on this type of cell [13,14].

In vitro studies with chitosan-based scaffolds have shown that it is a biopolymer capable of osteoconduction, in addition to supporting adhesion and proliferation of osteoblasts and the formation of mineralized bone matrix; chitosan’s highly versatile nature thus makes it attractive as a potential material for bone scaffolding [12,15].

However, one of its limitations is its low mechanical properties, which is why it is often physically or chemically crosslinked to provide better structural support [15,16].

Ammonium hydroxide (AH) has been used in tissue engineering to create scaffolds from gels with the proper porosity for cell proliferation [17]. Reyna et al. showed that physically crosslinking chitosan scaffolds with ammonium hydroxide improved the homogeneous morphology and porosity of the scaffolds [18].

Chitosan is a biopolymer that is soluble only in acidic media. Therefore, for the elaboration of the scaffolds, it is necessary to dissolve the chitosan in acids and, subsequently, eliminate the solvent from the scaffolds. For this, neutralization processes are carried out to regenerate the NH_2_ groups in chitosan and make the surface hydrophilic and biocompatible with the cells [19]. Chitosan as a scaffold has been widely studied; however, there is little evidence on the effects of neutralization and the benefits for the physicochemical, mechanical, and biological properties of crosslinked chitosan scaffolds. Hence, the present study assessed the effects of neutralization on chitosan scaffolds crosslinked with AH

## 2. Results and Discussion

### 2.1. Preparation of CHT–AH Scaffolds

**Figure 1:**
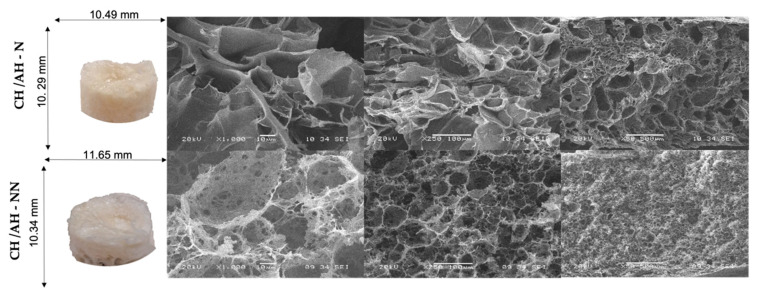
SEM surface morphology of neutralized (CHT/AH–N) and non-neutralized (CHT/AH–NN) CHT–AH scaffolds.

The scaffolds were obtained through physical crosslinking between CHT and ammonium hydroxide (Figure 1). Chitosan is insoluble in neutral and alkaline solutions but is soluble at a pKa of 6.5 due to its structure’s protonation of free amino groups. Neutralization of chitosan solutions at a pH value above 6.2 results in the immediate formation of a hydrated gel-like precipitate [20]. The sponges obtained showed a porous structure due to the lyophilization process [21].

Szymon Mania et al. reported that porosity and pore size (≈100 µm) at the macroscopic and microscopic levels are important parameters for biomedical applications and adequate cell proliferation, since it is considered that porosity contributes to ensuring the adequate exchange of nutrients or gases and cell proliferation. They also reported that their foams were yellowish-white, like those obtained in our study. This coloration can be attributed to the raw materials from which they were obtained or the chemicals used for foam preparation [22]. Chitosan gels crosslinked with ammonium hydroxide have a white color. Because they are physically crosslinked, they are readily solubilized in an aqueous solution of acetic acid, as reported in the literature. However, they are not soluble in distilled water [4,23].

The scaffolds’ elaboration was subjected to a freezing process prior to the lyophilization to improve the porosity. Freezing of a polymer solution causes a thermodynamic separation of the solution into polymer-rich and solvent-rich phases [24]. The polymer-poor phase’s subsequent growth and coalescence form the scaffolds’ pores when the solvent is removed during the lyophilization process.

Reyna et al. conducted a comparative study of two crosslinking methods (glutaraldehyde and ammonium hydroxide) in chitosan hydrogels containing collagen, observing that the chitosan scaffolds that were physically crosslinked exhibited a homogeneous morphology with greater porosity [18].

### 2.2. Physicochemical and Structural Characterization of CHT-AH Scaffolds

#### 2.2.1. Surface Morphology by SEM

Figure 1 shows the morphology of neutralized and non-neutralized CHT-AH scaffolds at 50, 250, and 1000× magnifications. The SEM images showed highly interconnected, porous morphology, as reported in previous studies [18,25].

The structure of our scaffolds showed sufficient porosity and interconnected pores, so they could be considered suitable to allow the nutrition, proliferation, and migration of cells [26]. In addition, the morphology of our materials, for all samples, showed a relatively large pore size similar to that described by Rungsima et al., who also used a low-molecular-weight chitosan [3].

The more homogeneous morphology was found to correspond to the neutralized scaffolds with smaller voids/pores and heterogeneously distributed pores. A similar microstructure and surface morphology were observed on non-neutralized scaffolds, but they appeared less homogeneous.

We assumed that the crosslinking effect of ammonium hydroxide influenced the chitosan structure through hydrogen bond formation. Reyna et al. reported that the non-porous areas may be due to the presence of closed pores produced by the contraction of the foam structure [18]. In our samples, this could be due to the additional drying that the foams experienced after neutralization with NaOH and washing with distilled water, where small pores in the CHT-AH scaffolds could be observed. Jia Yan et al. reported similar morphology in their chitosan foams; they found discontinuous lamellae incompletely wrapped by the chitosan matrix, along with some small aggregates [18,27,28].

The pores that were observed in our foams could be attributed to interactions of hydrogen and amide bonds. This type of interaction improved the stability of the material and resulted in the formation of a three-dimensional foam, with porosity favorable for cell growth [29].

Surface topography is an important factor that alters cell adhesion and distribution on the biomaterial surface [30]. The porosity of scaffolds depends on the ratio of their composition, the types of crosslinking (i.e., physical or chemical), the crosslinking agents used, and the manufacturing method. Evidence is still lacking to clarify whether scaffolds with uniform pore size distribution are more efficient in tissue regeneration than those with variable pore size distribution. The distribution and morphology of the pores are designed according to their application. For example, in bone tissue engineering, a small pore size (<100 mm) has been reported to be associated with the formation of unmineralized fibrous or osteoid tissue. A pore size of up to 800 mm is appropriate to provide adequate space for cell growth. Moreover, previous studies have shown that pore size < 10 mm creates a larger surface area that stimulates greater ion exchange and bone protein adsorption [29].

Regarding pore size, a larger pore is preferred for cell growth and proliferation, since the pores will occlude later than smaller pores during progressive growth and, consequently, provide an open space for growth, supplying nutrients and oxygen [29].

In agreement with this, Nitar et al. [31] and Karimi [32] reported that chitosan scaffolds produced by freeze-gelation increased the growth and proliferation of pulposus cells of human intervertebral disks, attributing these characteristics to the formation of pores of various sizes, and concluded that the freeze-gelation process is a suitable method for fabricating various chitosan-based composite biomaterials for cell proliferation.

#### 2.2.2. Elemental Composition by EDX

The results of the EDX analyses are shown in Table 1. The main difference between the neutralized and non-neutralized scaffolds was observed in the percentage of nitrogen obtained in the non-neutralized foams (7% at.), whose pH was 10.2, compared to the neutralized ones (12% at.) with a pH value of 7. This effect can be explained because incorporating the NaOH salt in chitosan modulates electrostatic and hydrophobic interactions and hydrogen bonds. It has been reported that the salts can cause three main effects: (a) increasing the pH, (b) inhibiting the immediate precipitation of the hydrated gel, and (c) imparting thermo-gelling characteristics at 37 °C. The addition of the neutralizing solution could cause reductions in electrostatic repulsion and increases in hydrogen bonds between chitosan chains [20].

#### 2.2.3. Fourier-Transform Infrared (FTIR) Spectroscopy

**Figure 2:**
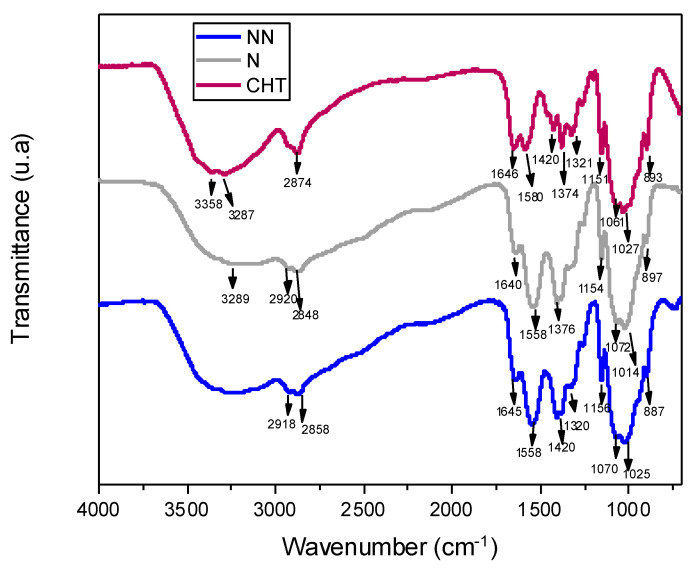
FTIR spectra of pristine CHT, neutralized AH-crosslinked CHT (N), and non-neutralized AH-crosslinked CHT (NN).

Figure 2 shows the spectrum of the pristine chitosan, showing bands at 3358 cm^−1^ corresponding to the O–H groups, while amine group (N–H) symmetric strain vibration characteristics are observed at 3287 cm^−1^. The bands at 2924 cm^−1^ and 2874 cm^−1^ are attributed to –CH_2_ group stretching vibrations correlated with pyranose rings [33]. Bands located at 1646 cm^−1^ correspond to –C=O stretching of the amide group [33], while bands at 1580 cm^−1^ correspond to amide II [3], with the same intensity as the previous amide I band [34,35].

The infrared spectra of the non-neutralized ammonium-hydroxide-crosslinked CHT scaffolds (CHT-AH NN) exhibited bands at 3350–3280 cm^−1^ corresponding to stretching vibrations of OH and NH stretching, and at 2918 and 2858 cm^−1^ due to C–H bond stretching. NH_2_ bending in amide I at 1645 cm^−1^ and N-H bending in amide II at 1558 cm^−1^ changed their intensity ratio, i.e., during crosslinking, amide I was reduced. The band located at 1424 cm^−1^ corresponded to C–H vibrations, whereas the peak at 1370 cm^−1^ was attributed to methyl vibrations in the acetamide group, and the 1320 cm^−1^ band was associated with amide III. The skeleton vibrations typical of the chitosan structure appeared at 1070–1025 cm^−1^ [30]

The existence of a large number of free COOH groups related to unreacted acetic acid can be characterized by a strong peak at 1730 cm^−1^ [36]. However, this was not observed in our samples. The characteristic bands of amide I, amide II, and amide III were found at 1637 cm^−1^, 1548 cm^−1^, and 1317 cm^−1^, respectively [37,38]. Neutralization shifted the amino II band from 1580 cm^−1^ in the as-prepared CHT to 1558 cm^−1^ but did not change in intensity or peak position compared to the non-neutralized scaffold. The band located at 1158 cm^−1^ was assigned to the C–O–C of the glycosidic bond, and skeletal vibrations typical of the chitosan structure were observed at 1014 cm^−1^. The peaks at 1377 cm^−1^ and 1420 cm^−1^ were assigned to the methyl symmetric deformation mode. Under acidic conditions, due to the excess of H+, a greater protonation of the amine and carbonyl groups on the oxygen is expected, with the consequent reduction in the bands. This is more evident as it approaches the dry state due to water’s antagonistic “deprotonation” effect. The hydroxyl groups of chitosan most exposed to solvents tend to form hydrogen bonds with water molecules rather than OH–OH or NH–OH bonds within the polysaccharide. It can be affirmed that the link with ammonium hydroxide occurs only physically, giving rise to the interactions within the gels and composite foams [39].

#### 2.2.4. Raman Spectroscopy

**Figure 3:**
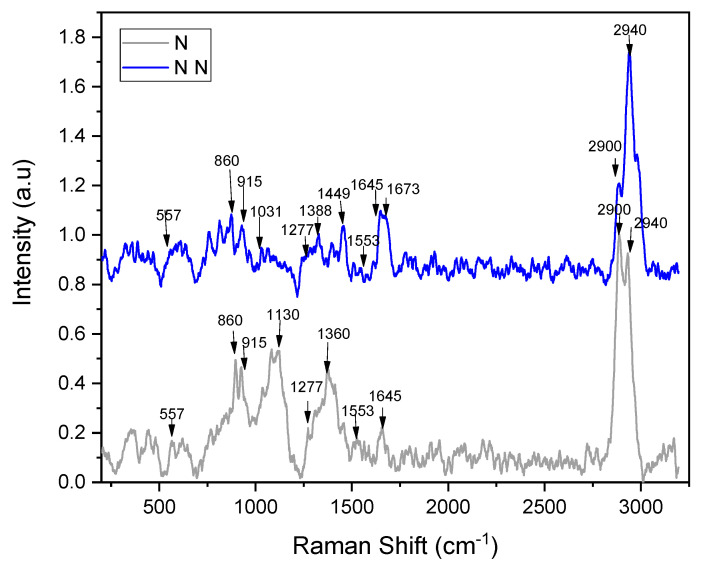
Raman spectra of neutralized (N) and non-neutralized (NN) AH-crosslinked CHT.

The characteristic bands of the non-neutralized chitosan scaffold (Figure 3) were observed at 2940 cm^−1^ (high intensity), 2900 cm^−1^, 1645 cm^−1^ (medium intensity) with a shoulder at 1673 cm^−1^, and low-intensity peaks at 1553 cm^−1^, 1449 cm^−1^ (medium intensity), 1388 cm^−1^ (low intensity), 1277 cm^−1^, 1031 cm^−1^, 915 cm^−1^, 860 cm^−1^, and 557 cm^−1^. In the neutralized scaffolds, the peak at 1645 cm^−1^ reduced its intensity, the peak at 1360 cm^−1^ was more intense, and another appeared at 1130 cm^−1^.

It has been reported that when using a 1064 nm laser, chitosan shows absorption bands at 2885 cm^−1^, 1419 cm^−1^, 1376 cm^−1^, 1116 cm^−1^, 900 cm^−1^, 491 cm^−1^, and 424 cm^−1^ [40]. Only some of these vibrations were observed with the 633 nm laser. Absorptions in the 2800–3000 cm^−1^ interval are derived from CH and CH_2_ or even CH_3_ bond stretching [41], whereas those in the 1000–1200 cm^−1^ interval indicate ẟ (C–C) and C–O–C band stretching [42]. The peaks at 1449 cm^−1^ and 1388 cm^−1^ can be assigned to the bending of CH groups in the pyranosidic skeleton. However, they have sometimes been associated with C–O–H stretching bonding, as reported by Schauenberg et al. [43].

The absorption of amide I observed at 1645 cm^−1^ and the band at 1553 cm^−1^ were of low intensity in Raman spectra in contrast to those observed in the infrared spectra, demonstrating that crosslinking with ammonium hydroxide was occurring. Interestingly, when the scaffold was neutralized, a split appeared in the signal at 2900 cm^−1^ and 2940 cm^−1^. On the other hand, the absorptions at 1360 cm^−1^ and 1130 cm^−1^ were observed to be more intense.

#### 2.2.5. Thermogravimetric Analysis (TGA)

The thermal stability of the sample was evaluated by determining the starting temperature of the degradation stage of the ammonium-hydroxide-crosslinked chitosan scaffolds and the maximum temperature during decomposition.

**Figure 4:**
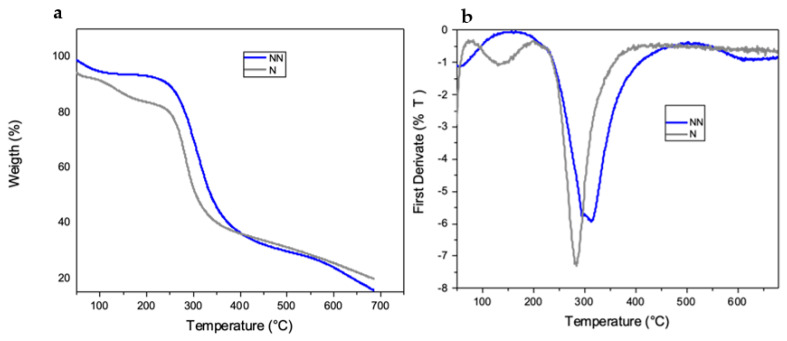
(**a**) Thermogravimetric analysis (TGA) and (**b**) DTGA thermograms of neutralized (N) and non-neutralized (NN) AH-crosslinked chitosan.

Figure 4 shows the TGA thermograms of the chitosan foams. In the neutralized NH_4_OH-crosslinked chitosan scaffolds, the decomposition temperature (Td) was observed at 285 °C (Table 2). On the other hand, the decomposition temperature of the non-neutralized NH_4_OH-crosslinked chitosan foam was observed at 316 °C (Table 2). These results suggest higher thermal stability in non-neutralized foams as the regenerated amino groups (from NH_4_OH treatment) and acetate salts render them more stable—possibly because of the effects of the electrostatic interactions on the structure [35]. Studies performing thermogravimetric measurements under non-oxidizing conditions (e.g., nitrogen atmosphere) suggested that the chitosan sample contains approximately 10% of the adsorbed water, which evaporates at low temperatures (i.e., below 100 °C), which means that the water is physically adsorbed and/or is loosely bound to the chitosan molecules (stage 1). The decomposition accompanying the subsequent 10% weight loss starts above 100 °C and reaches the maximum rate at 168 °C (stage 2). Typical water with strong hydrogen bonds is released, possibly as a consequence of the evaporation of ammonium and acetic acid on the scaffold [44].

The predominant stage of thermal degradation of low-molecular-weight chitosan occurs at a temperature range of 230–400 °C, with a 43% drop in the chitosan mass. This is caused by the depolymerization of the chitosan chains, the decomposition of the pyranose rings by dehydration and deamination and, finally, the opening reaction of the ring [16,45]. Additionally, the heat resistance is known to depend on the polysaccharide structure [46].

### 2.3. Compression Mechanical Test

The elastic modulus and compressive strength were determined by a mechanical compression test. The elastic modulus at compression (Ec) of the scaffolds was calculated in the elastic deformation zone of the foams, between 10 and 15% deformation in the strain–stress plot; the compressive strength (σ 10) of the foams was calculated at 10% deformation in all samples, as established in ASTM D1621 [47]. Table 3 reports the elastic modulus and compressive strength of the ammonium-hydroxide-crosslinked chitosan foams. The results indicate that the samples with the highest elastic modulus were the non-neutralized crosslinked chitosan scaffolds. The values obtained for the chitosan foams crosslinked with NH_4_OH were similar to those obtained by Qin et al. in 2019, who reported low-molecular-weight chitosan foams with an elastic modulus of 19 kPa [44]. For bone tissue engineering, a requirement is that the scaffolds have similar mechanical properties to the bone under repair to ensure their mechanical integrity [30]. Although this study reports values that are inferior to those exhibited by either trabecular or cortical bone, it also provides evidence of the deleterious effect of neutralization.

A scaffold for biomedical applications should provide a mechanical strength in the range of 50 to 350,000 kPa [3]. From the point of view of mechanical behavior, studies indicate that load transfer will be favorable when the modulus of elasticity between the implant and the bone tissue is similar. The modulus of elasticity of alveolar bone has been reported to be 20 GPa [48]. Therefore, in our study, the most suitable sample for the peri-implant bone tissue was the chitosan foam crosslinked with ammonium hydroxide with an elastic modulus of 19.1 kPa [46], considering that the DPSCs will produce the required bone.

Student’s *t*-test was performed to compare the elastic modulus and compressive strength between the two materials, and significant differences were found (*p* = 0.011) in the comparison of the elastic modulus between the neutralized and non-neutralized crosslinked chitosan scaffolds. The analysis found no significant differences between the materials for the compressive strength at 10% strain (*p* = 0.176).

### 2.4. Evaluation of Cell Viability and Proliferation on the Scaffolds

Cytotoxic effects can prevent the in vivo integration of a biomaterial by modifying the natural assimilation process [49]. Therefore, it is necessary to evaluate biomaterials’ cytotoxicity.

**Figure 5:**
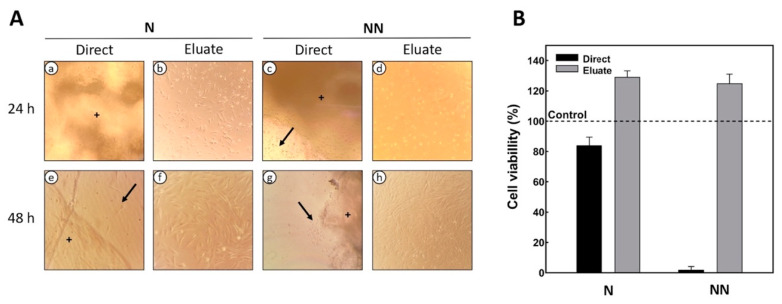
Microphotographs (inverted light) and viability of cultured DPSCs following direct or indirect contact with chitosan/AH. (**A**) Phase-contrast micrographs of DPSCs with direct or eluate contact at 24 and 48 h on neutral (N) and non-neutralized or alkaline (NN) scaffolds over 48 h. (**B**) MTT assay of DPSCs’ viability on N and NN scaffolds after 48 h of culture. The arrows point to the cells attached to the culture surface; +: sponge fragment in the microscope field.

The viability of DPSCs grown on 2D or 3D (direct contact) chitosan scaffolds under varying neutralization regimens was assessed using the MTT assay. In the direct cytotoxicity test, it was observed that after 24 h the cells expressed adhesion and a fibroblastic morphology in the neutralized chitosan. However, there were physical changes in the chitosan structure, making the scaffolds translucent (Figure 5A(a,e)). At the end of the evaluation time (48 h), the cells adhered to the biomaterial, and more significant confluence was observed, showing that the cellular density increased over the examination period. In contrast, non-neutralized scaffolds did not allow cell adherence and proliferation (Figure 5A(c,g)), while the cell viability decreased by 90% (Figure 5B). We assumed this effect to be the result of excess ammonium hydroxide, which in large amounts is considered to be toxic to cells and was also trapped between the pores of the scaffold, creating a less viscous layer (or surface) to which the cells were unable to adhere. The presence of focal adhesions is directly or indirectly related to the nature and the amount of the surface adsorbed [19]. In our study, only neutralized scaffolds developed focal adhesions.

Indirect cytotoxicity tests, after 24 h, showed that cells on the neutralized chitosan scaffolds (N) had higher proliferation (Figure 5B) compared to those on the non-neutralized chitosan (NN) (Figure 5A(d)). After 48 h, higher cell confluency was observed for the indirect treatments on both scaffolds (Figure 5A(f,h)), and the viability was unchanged (Figure 5B). The cell viability of the scaffolds was higher than 80%, indicating their good cytocompatibility.

Intracellular (pHi) and pericellular (pHe) pH are considered to be influential factors in normal cell function, knowing that changes in their values affect signaling functions. Mammalian cell proliferation performs best at a permissive pH in the slightly alkaline range (7.0–7.2). [50]. Cell proliferation is known to be strongly affected by pH. However, many questions remain about whether pH alone can play a driving/signaling role in driving cell proliferation [50].

Noriega et al. reported that treatment with different concentrations of NaOH in chitosan scaffolds affected cell proliferation and differentiation [19]. The polyelectrolytic effect of chitosan in acid solutions is determined by the chelating capacity of the amino groups. In acidic solutions, amino groups are protonated to NH_3_^+^ with a pKa value of 6.1 to 6.4. When exposing the chitosan acetate surface in contact with a sodium hydroxide solution, the acetate ions associate with their counterion (Na^+^), and the NH_3_^+^ loses a proton, forming a water molecule, which generates a microenvironment on a biomaterial surface with the characteristics of a polyelectrolyte. In addition, it has also been reported that higher viscosities are rendered at lower pH, while lower viscosities occur at higher pH (0.1 M) [19]. In our scaffolds, the more viscous surface showed a detrimental effect on cell proliferation; this may have been due to the poor adhesion of the cells on the scaffold.

## 3. Materials and Methods

### 3.1. Materials

Chitosan (CHT)—with a low molecular weight of 183–364 g/mol^−1^ (calculated previously [18]) and a degree of deacetylation of 75–85% (batch number: STBF3282V provided by the supplier)—and ammonium hydroxide (AH, 28% NH_3_ in H_2_O ≥99.99% trace metals basis) were purchased from Merck (Saint Louis, MO, USA). Acetic acid was provided (AA, ≥99.5% purity) by J.T. Baker (Mexico). The 3-4,[5-dimethylthiazol-2-yl]-2,5-diphenyl tetrazolium bromide cell viability assay (MTT assay) was obtained from Merck (Saint Louis, MO, USA). Eagle’s Minimum Essential Medium (MEM), fetal bovine serum, and penicillin/streptomycin were purchased from Sigma-Aldrich (St. Louis, MO, USA).

### 3.2. Methods

#### 3.2.1. Scaffold Preparation

Chitosan–ammonium hydroxide scaffolds were prepared as reported in a previous work [18]. Briefly, the CHT solution was prepared by dissolving 1.5 g of CHT in 0.4 M acetic acid (20 mL). The mixture was stirred overnight with a magnetic stirrer at room temperature (25 °C) to ensure complete dissolution (the final pH of the solution was 4.5). Subsequently, the resulting solution was deposited in a 24-well cell culture plate, leaving spaces between wells. Afterward, 2.5 mL of 1N ammonium hydroxide (12.8 mL of NH_4_OH per liter of water) was poured into the empty spaces of the culture dish. The 24-well cell culture plate containing the solutions was placed in a hermetically sealed container inside a fume hood. Physical crosslinking (i.e., gelation) was induced by ammonia diffusion for 48 h, as reported by Reyna et al. [18].

#### 3.2.2. Neutralization Procedure of Chitosan–AH Scaffolds

Half of the obtained hydrogels were further neutralized with a sodium hydroxide (NaOH) solution (5% by weight) for 15–20 min. Subsequently, they were copiously rinsed with distilled water to remove ammonium acetate and excess NaOH; this procedure was repeated until reaching a neutral pH (~7.0 to ~7.2). The other half of the hydrogels were kept at the original pH (pH: 10.2) after crosslinking with AH. In order to obtain 3D scaffolds, hydrogels with (N) and without the neutralization treatment (NN) were refrigerated for 24 h, and then they were dried in a LABCONCO 4.5 FreeZone freeze-dryer at −50 °C and a pressure of 0.018 mBar for five days.

#### 3.2.3. Characterization of Chitosan–AH Scaffolds

The morphology and pore size of the scaffolds were observed on a JEOL JSM-6360 LV scanning electron microscope (SEM) (Akishima, Tokyo, Japan) operating at 20 kV; each sample was coated with a thin layer of gold using a Denton Desk II Sputter Coater (Moorestown, NJ, USA). Energy-dispersive X-ray spectroscopy (EDX) (Oxford Instruments, INCA X-Sight 7582, High Wycombe, UK), coupled with the microscope, was used to determine the elemental composition. The chemical structures of the scaffolds were studied using an FTIR spectrophotometer (Nicolet Thermo-Scientific 8700, Waltham, MA, USA) with the attenuated total reflectance (ATR) technique. Spectra were collected over 4000 to 600 cm^−1^ with a zinc selenide crystal. The average number of scans was 100, with a resolution of 4.0 cm^−1^, and corrected for H_2_O and CO_2_. Raman determination was performed using the InVia™ Raman Renishaw microscope (Wotton-under-Edge, Gloucestershire), with a laser of 633 nm, a power of 50% analyzed in the spectral interval between 200 and 3200 cm^−1^, a power of 100%, grid of 1800, and an objective of 50× with an exposure time of 60 s. The thermogravimetric analysis was carried out using a TGA 8000™ from PerkinElmer (Waltham, MA, USA), in the temperature range 45–750 °C and under an inert atmosphere (i.e., nitrogen) at a heating rate of 10 °C/min.

#### 3.2.4. Mechanical Analyses

Compression tests were performed according to ASTM D 1621-00. The specimens used (n = 5) were cylinders of 5.0 ± 0.3 mm in height and 6.0 ± 0.2 mm in diameter. The tests were performed on a Mini-Shimadzu AG-1 universal testing machine at room temperature (25 °C), using a 1 kN load cell, at a crosshead speed of 1 mm/min. The compressive strength (σ, MPa) was calculated using the following equation:

4*F*σ = *πD*2

where *F* is the maximum applied load (N) and *D* is the specimen’s diameter (mm). The results obtained from the mechanical test (compression) were statistically analyzed using Student’s *t*-test; *p*-values of <0.05 were considered statistically significant.

#### 3.2.5. Cell Viability and Proliferation Studies

##### Direct Contact

DPSCs were seeded on 12-well plates at a density of 10^4^ cells/well in MEM-alpha medium, 10% (*v*/*v*) fetal bovine serum, and 1% (*v*/*v*) antibiotic/antifungal solution. Plates were left for 24 h in the incubator at 37 °C, in a 5% CO_2_ atmosphere, to adhere to the wells. After sterilization by UV-C light for 15 min, the materials to be tested were placed in the center of each well and on top of the adhered cells.

##### Indirect Contact

Indirect contact assays were conducted according to the practical guide for preparing samples and reference materials (ISO 10993-12) [51]. For this, 10 mg of chitosan was placed in contact with 10 mL of MEM-alpha for 24 h at 24 °C. After this, the extracts were centrifuged and filtered with a 0.22 µm Spritzer TPP^®^ syringe filter. For the eluate tests, extracts replaced the culture medium in a 12-well plate with 104 cells/well. The seeded cells were incubated at 37 °C, 5% CO_2_, and 95% RH for 48 h. DPSCs grown in the plates without extracts were used as 2D controls. All samples were checked daily using a Nikon Eclipse E600 inverted light phase-contrast microscope. For both techniques (i.e., direct and indirect), positive (i.e., cells with only the culture medium) and negative (i.e., cells with hydrogen peroxide) controls were included, which were also targeted with MTT solution.

## 4. Conclusions

Based on the results obtained, it can be concluded that there was a physical crosslinking of chitosan with ammonium hydroxide, which allowed the formation of chitosan gels in a hermetic environment. Later, the formation of foams after lyophilization was confirmed by FTIR, where slight changes in the amide ratio of the scaffolds were detected. The porosity of the scaffolds was corroborated by scanning electron microscopy. The compression test indicated that the neutralized NH_4_OH-crosslinked chitosan scaffolds had lower elastic moduli than the non-neutralized scaffolds. The cell viability tests with DPSCs showed that the scaffolds produced from chitosan crosslinked with NH_4_OH were non-toxic and biocompatible at alkaline and slightly alkaline pH, suggesting that this biomaterial has potential for tissue engineering applications in the biomedical field.

## Figures and Tables

**Table 1:** EDX elemental composition of neutralized and non-neutralized chitosan/AH.

Scaffolds	%C	%O	%N	%Na
Neutralized	48 ± 5	38 ± 2	11 ± 4	3 ± 1
Non-neutralized	51 ± 5	41 ± 1	7 ± 4	1 ± 1

**Table 2:** Decomposition temperatures and weight loss of neutralized (N) and non-neutralized (NN) AH-crosslinked chitosan.

Scaffolds	T_d_ (°C)	T (°C) at 50% Weight Loss
Neutralized	285	278
Non-neutralized	316	263

**Table 3:** Scaffolds’ mechanical properties under compression.

Scaffolds	Elastic Modulus (Ec) kPa	Compressive Strength (σ 10) kPa
Neutralized	8.8 ± 1.2	99.2 ± 15.7
Non-neutralized	19.1 ± 0.9 CV 4.7%	127.0 ± 10.9 CV 8.6
*p*-Values of *t*-test	0.011 *	0.176

* Statistically significant.

## Data Availability

The data presented in this study are available upon request from the corresponding author. The data are not publicly available due to its content being a thesis from a bachelor’s degree in dentistry.
